# Establishing a mucosal gut microbial community *in vitro* using an artificial simulator

**DOI:** 10.1371/journal.pone.0197692

**Published:** 2018-07-17

**Authors:** LinShu Liu, Jenni Firrman, Ceylan Tanes, Kyle Bittinger, Audrey Thomas-Gahring, Gary D. Wu, Pieter Van den Abbeele, Peggy M. Tomasula

**Affiliations:** 1 Dairy and Functional Foods Research Unit, Eastern Regional Research Center, Agricultural Research Service, US Department of Agriculture, Wyndmoor, Pennsylvania, United States of America; 2 Division of Gastroenterology, Hepatology, and Nutrition, Children’s Hospital of Philadelphia, Philadelphia, Pennsylvania, United States of America; 3 Perelman School of Medicine at the University of Pennsylvania, Philadelphia, Pennsylvania, United States of America; 4 ProDigest, Ghent, Belgium; National Institute for Agronomic Research, FRANCE

## Abstract

The Twin Simulator of the Human Intestinal Microbial Ecosystem (TWINSHIME^®^) was initially developed to study the luminal gut microbiota of the ascending (AC), transverse (TC), and descending (DC) colon regions. Given the unique composition and potential importance of the mucosal microbiota for human health, the TWINSHIME was recently adapted to simulate the mucosal microbiota as well as the luminal community. It has been previously demonstrated that the luminal community in the TWINSHIME reaches a steady state within two weeks post inoculation, and is able to differentiate into region specific communities. However, less is known regarding the mucosal community structure and dynamics. During the current study, the luminal and mucosal communities in each region of the TWINSHIME were evaluated over the course of six weeks. Based on 16S rRNA gene sequencing and short chain fatty acid analysis, it was determined that both the luminal and mucosal communities reached stability 10–20 days after inoculation, and remained stable until the end of the experiment. Bioinformatics analysis revealed the formation of unique community structures between the mucosal and luminal phases in all three colon regions, yet these communities were similar to the inoculum. Specific colonizers of the mucus mainly belonged to the Firmicutes phylum and included *Lachnospiraceae* (AC/TC/DC), *Ruminococcaceae* and *Eubacteriaceae* (AC), *Lactobacillaceae* (AC/TC), *Clostridiaceae* and *Erysipelotrichaceae* (TC/DC). In contrast, *Bacteroidaceae* were enriched in the gut lumen of all three colon regions. The unique profile of short chain fatty acid (SCFA) production further demonstrated system stability, but also proved to be an area of marked differences between the *in vitro* system and *in vivo* reports. Results of this study demonstrate that it is possible to replicate the community structure and composition of the gut microbiota *in vitro*. Through implementation of this system, the human gut microbiota can be studied in a dynamic and continuous fashion.

## Introduction

It is well recognized that there is a link between diet and the gut microbiota, and that the human gut microbiota has an impactful effect on both health and disease [1–8]. However, the most compelling reports have been based on *in vivo* studies that relied heavily on data derived from fecal samples, which cannot be used to describe the activities of the gut microbiota in the different colon regions, or from the luminal or mucosal phases of the gastrointestinal tract (GIT) [9–12]. Also, the strong interplay between the host cells and the resident microbes makes it nearly impossible to distinguish between the two using an *in vivo* model. Thus, *in vitro* research using GIT simulators is not only relevant, but an essential complement to *in vivo* studies.

Considerable efforts have been made to develop a GIT simulator that allows the *in vitro* experiments to be conducted in an environment mimicking the *in vivo* physiological conditions. Culture systems from a single vessel to those consisting of multi-compartments to maintain continuous operational conditions have been evaluated [10,13–18]. The Twin Simulator of the Human Intestinal Microbial Ecosystem (TWINSHIME^®^, ProDigest; Ghent, Belgium) is the most recent outcome of this developmental process [19,20]. It was designed as a system capable of reproducing the conditions of the human GIT, with the purpose of studying the gut microbiota of the colon. It divides the colon into the ascending (AC), transverse (TC), and descending (DC) regions, and has computer controlled, automatic feeding of the system three times a day.

The TWINSHIME distinguishes itself from other simulators by adding a mucosal surface to each region (ofter referred to as M-SHIME), thus research on bacterial behavior in the mucin-phase of the gut is not only available, but also can be implemented simultanously with that of the luminal-phase [21–23]. The addition of a mucosal surface is an important development for *in vitro* studies, because the the mucosal surface is colonized by a microbiota community distinct from the luminal community, and displays specific functionalities [4,5]. Moreover, given its proximity to the host epithelium, the mucosal microbiota is considered to have a greater potential to affect human health.

Previous reports have demonstrated that the TWINSHIME is capable of reproducing a stable human gut microbial community [14,19,20] 2–3 weeks after inoculation and, that the diversity of the community is similar to the inoculum with differences in community structure between the three colon regions [19]. However, these reports lack an in-depth and technologically advanced analysis on the community structure of the individual colon regions compared to the original inoculum and to each other. Specifically, these reports only disucss the microbiota of the luminal content, and do not address the mucosal microbiota. Earlier work looking at the mucosal phase in a TWINSHIME set up, revealed that the *in vitro* mucosal microbiota was representative of an *in vivo* community and preserved inter-individual specificities of the five human donors tested [22]. Yet the conclusions drawn from this report were limited, because the experiment focused only on a single colon region (transverse colon) during a short time period (3 days), and determined the microbiota composition using a HITChip microarray, which is a targeted sequencing method [22].

Importantly, this previous work did not look at the stability of the *in vitro* mucosal microbiota over time, or the ability for the mucosal microbiota to develop region specific communities. Both of which are key points that need to be addressed for two primary reasons. First, the mucus layer is regularly renewed *in vivo*; *in vitro* this is simulated by physically replacing the mucosal surface. However, this type of replacement may result in instability, since it does not mimic the continuous replacement that occurs *in vivo*. Second, the human colon *in vivo* harbors unique communities that are region specific. Region specificity for the mucosal surface must be demonstrated in the *in vitro* model.

In the present study, a gut microbial community was established and maintained for six weeks in a TWINSHIME apparatus, containing a mucosal surface. The community composition of the microbiota from both the luminal and mucosal phases of each colon region were compared to the community from the donor’s fecal sample, and to each other by 16S rRNA sequencing, an untargeted sequencing method. Both the community dynamics and SCFAs produced from each region were analyzed over time and compared intra-regionally. The results of this study were used to demonstrate (i) stability and (ii) colon-region specificity of not only the luminal, but importantly, also the mucosal microbiota at the level of community composition and microbial activity (SCFA production). An in depth understanding on the differentiation of each individual region and phases of the system is important, first, because the priniciple reason for using an *in vitro* system is that it allows for site specific studies on the gut microbiota. Therefore, these sites need to be fully defined. Second, there is an increasing body of evidence showing that not only are the bacterial communities in the luminal and mucosal phases unique, they also have different functions [4,5]. Last, understanding the differences and similiarities between these site specific communities will allow for targeted applications of both diet modifications and therapeutic approaches.

## Materials and methods

### Materials

The defined medium (DM) was purchased from ProDigest (Ghent, Belgium). It contained (in gL^-1^) arabinogalactan (1.2), pectin (2.0), xylan (0.5), resistant starch (4.0), glucose (0.4), yeast extract (3.0), peptone (1.0), mucin (3.0), and cysteine (0.5). The defined medium was adjusted to pH 2 prior to autoclaving. Pancreatic juice contained (in gL^-1^) NaHCO_3_ (12.5) (Sigma-Aldrich, Saint Louis, MO), bile salts (6.0) (BD, Franklin Lakes, NJ), and pancreatin (0.9) (Sigma-Aldrich, Saint Louis, MO). Mucin-Agar containing carriers were prepared by dipping plastic, hollowed carriers (DI, 5 mm; ProDigest) into a mucin-agar solution. The Mucin-Agar solution was prepared by boiling 1% bacterial agar in autoclaved MilliQ water three times, to dissolve 5% type II porcine mucin (Sigma-Aldrich). The filled carriers were allowed to solidify under laminar flow in a biosafety cabinet at room temperature and stored at -4 °C until use.

Fecal samples (Microbiome Health Research Institute; Boston, MA) were harvested from an individual American, randomly selected from a pool of typical, Western diet consumers, between the ages of 21 and 45 years old, with an average Body Mass Index (BMI), who had been antibiotics-free for at least 1 year. According to the provider, the donor of the fecal sample was required to fill out a medical history questionnaire and interviewed in a process similar to that used for blood donors. A series of tests were performed on both fecal and blood samples to screen for any potentially infectious pathogens before deposition of fecal material. Sixty days post donation, a second round of tests were performed on both the fecal and blood samples. Upon both sets of tests proving negative for the presence of pathogens, the fecal sample thus collected was homogenized to make a 10% feces in glycerol buffer solution. The homogenate was then aliquoted into 250 mL containers, frozen, and stored at -80°C (http://www.Openbiome.org, cited December 01, 2016). The same homogenate was used to inoculate both systems, simultaneously, for each experiment.

### TWINSHIME set up and operation

The TWINSHIME system was manufactured by ProDigest (Ghent, Belgium) and assembled at the USDA-Eastern Regional Research Center. The apparatus is composed of two completely independent SHIME systems, both containing five bioreactors set up in sequence to represent the stomach (ST), small intestine (SI), ascending colon (AC), transverse colon (TC), and descending colon (DC) (S1 Fig). The systems were maintained at 37°C by a water jacket, and anaerobic conditions sustained by sealing the vessels and applying sterile nitrogen flow. The pH of each bioreactor was computer controlled to match physiological conditions of the colon regions using 0.5 M HCl and 0.5 M NaOH, with the pH values of 2 for the ST, 6.6 for the SI, 5.6 for the AC, 6.15 for the TC, and 6.6 for the DC [18,19].

In the current experiment, the bioreactors of both SHIME systems contained defined media at a volume of AC (500 ml), TC (800 ML), and DC (600 ml) [18,19]. Each colon region of one system (SHIME 1) also contained 60 mucin carriers, while the second system (SHIME 2) had the luminal phase only. The mucosal carriers provide a mucin surface for bacterial to grow. In this way, the mucosal surface of the intestines can be simulated. The systems were fed three times a day to mimic physiological conditions, with 140 ml defined media and 60 ml pancreatic juice containing bile salts, as previously described [18,19]. During the feeding cycle, the same volume of fluid was transported through the system simultaneously. The flow of fluid was from the SI to the AC, AC to the TC, TC to the DC, and then DC out to waste, as described in the operation menu provided by the manufacturer.

The SHIME system was fed three time a day on a fixed time schedule, which provides the community with fresh nutrients, which are then metabolized. This increase, and then decrease, in metabolism means that there is some fluctuation in metabolites on a daily basis. In other words, the composition and ratio of the microbial community, as well as their metabolite levels, are dependent on the point of the feeding cycle. In the current study, every sampling was performed at the same point in the feeding cycle, thus, the comparison of data obtained from one sampling with that from the following sampling is meaningful.

Twice a week, 60 minutes prior to feeding, slurry-like samples were taken from each colon region of the two systems, and 30 mucin carriers were removed from each colon region of SHIME 1 and replaced with 30 new mucin carriers. For luminal fluid, the samples were centrifuged at 5000g for 10 mins at 4°C. The supernatant was filtered through a 0.22μm PES filter to make bacterial free supernatant (BFS) and stored at -80 °C for SCFA analysis, and the bacterial pellet was stored at -80°C for DNA extraction. For mucosal samples, the harvested mucin was aliquoted into tubes at a volume of 0.25–0.5g and stored at -80°C until needed. All experiments were performed in triplicate to confirm the results. Samples harvested from days 0, 1, 3, 10, 15, 23, 30, and 43 post inoculation were sent for 16S rRNA DNA sequencing and samples from days 0, 1, 3, 6, 10, 15, 20, 23, 27, 30, 34, 38, 41, and 43 post inoculation were used for SCFA analysis.

### DNA sequencing and analysis

The DNA extraction was performed using the CTAB DNA extraction method, as described previously [24]. The DNA extracts were quantified using a nanodrop and stored at -80°C until needed. In order to determine the community composition at each time point, in each reactor, extracted DNA was sent for NextGen, 16 Small Ribosomal RNA (16S RNA) sequencing of the V1V2 region using the MiSeq Illumina platform [24].

The sequencing was performed at the Microbiome Center/Children’s Hospital of Philadelphia (CHOP), and data were processed using QIIME software version 1.9, a widely used analytical pipeline [25]. Reads pairs from 16S rRNA marker gene sequencing were joined to form a complete amplicon sequence for the V1-V2 region, with a minimum overlap of 35 base pairs and a maximum overlap difference of 15%. Sequences were filtered to remove low quality reads, with a minimum quality threshold of Q20. Reads were clustered at 97% sequence identity using UCLUST v. 1.2.22 to form Operational Taxonomic Units (OTUs) [26]. Taxonomy was assigned to the sequences using the Greengenes reference database v. 13_8 [27,28]. Similarity between samples was assessed by weighted and unweighted UniFrac distances [29,30]. The richness measure was calculated by counting the number of unique OTUs at 50,000 read sequencing depth. Within each sample, the OTU counts were divided by the total OTU count to obtain the relative abundances. The relative abundances of the OTUs that got assigned to the same bacterial family was summed to get family level relative abundances. The abundances were then log transformed and compared between the bioreactors using 2 tailed student *t-*tests. The statistical comparisons were based only on the relative abundance at the family level for the stable community, and therefore included data from five time points, days 10, 15, 23, 30, and 43. The p values were adjusted for false discovery rate using the method of Benjamini and Hochberg [31].

### SCFA analysis

For SCFA analysis, standards were made using analytical grade chemicals that were purchased from Sigma-Aldrich (Saint Louis, MO, USA). They are linear SCFAs, such as acetic acid, propionic acid, butyric acid, valeric acid, and caproic acid; as well as branched SCFAs (BSCFAs), such as isobutyric acid (2-methylpropanoic acid), isovaleric acid (3-methylbutanoic acid), isocaproic acid (4-methylpentanoic acid), and 2-methyl hexanoic acid. Standard curves were made at the concentrations ranging from 5 to 2,500 ppm for each individual chemical.

The frozen BFS samples were thawed at 40°C for 30 minutes, and a fraction of the filtered sample was mixed with diethyl ether (1:1, v/v) for liquid-liquid extraction. The organic extracts were transferred to a GC/MS (Shimadzu QP2010 Ultra; Shimadzu, Columbia, MD) equipped with Stabilwax-DA column, 30m, 0.25mm ID, 0.25μm, (Restek Corporation, Bellefonte, PA, USA) [32] and run for SCFA measurement using the following settings: Initial column temperature, 110°C, held for 1 min; increased to 220°C at 22°C/min, and then held at this temperature for 3 mins. For SCFA quantification, standard curves were constructed using individual SCFA standards and 2-Methyl hexanoic acid as the internal standard.

The average total SCFA is the summation of all SCFAs measured in each intestinal region after stabilization, and the average BSCFA is the summation of all branched SCFAs measured in each intestinal region after stabilization (Days 20, 23, 27, 30, 34, 38, 41, and 43). At each time point 3 x 1 ml samples were taken from each bioreactor, each sample was extracted three times, each extraction was measured three times on GC/MS. The statistical significance between the amounts of total SCFA and BSCFA in each region was determined using a 2-tailed student *t*-test based on the data from these time points.

## Results

### Establishing a stable luminal and mucosal gut microbial community using the TWINSHIME

**Fig 1:**
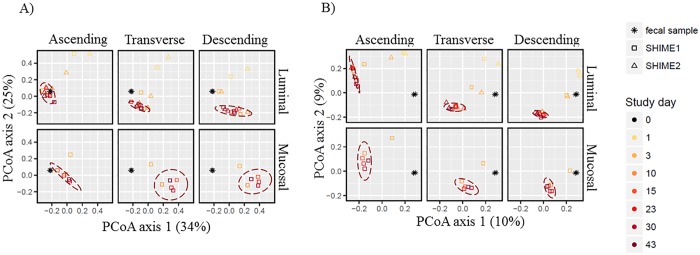
PCoA plot based on UniFrac distances of the gut microbial communities established in the luminal and mucosal phases for the ascending, transverse and descending regions over time. After calculating the axes, the samples are faceted into their corresponding regions. The dashed ellipses represent samples in that facet 3 days after inoculation that fall within a 95% confidence interval. (A) Weighted UniFrac distance analysis, (B) Unweighted UniFrac distance analysis.

The TWINSHIME system was developed in order to culture the gut microbiota of the large intestine for *in vitro* studies. One critical paramater is that the communities developed in this system reach a steady state. In order to substantiate stabilization, pairwise weighted and unweighted UniFrac distances were calculated from the 16S rRNA results and the bacterial communities were visualized by principal coordinate analysis (Fig 1). Both the weighted (Fig 1A) and unweighted PCoA (Fig 1B) demonstrate that clustering occurs after day 3, and that mature communities are established at day 10, indicating that a stable luminal and mucosal microbiota was achieved. The weighted PCoA (Fig 1A) demonstrated that the bacterial community established in the mucosal phase of each region was further in distance from the original sample than the community in the corresponding luminal phase. This difference was statistically significant, with p < 0.05 for all three regions based on a 2 tailed student *t-*test. In contrast to the weighted PCoA (Fig 1A), the unweighted analysis (Fig 1B) revealed that the bacterial community in the AC region is the furthest from the original sample. According to the weighted PCoA analysis, the average distance between the AC community, after stabilization, and the fecal sample was 0.32 ±0.04, whereas for unweighted this distance was 0.85±0.02.

**Fig 2:**
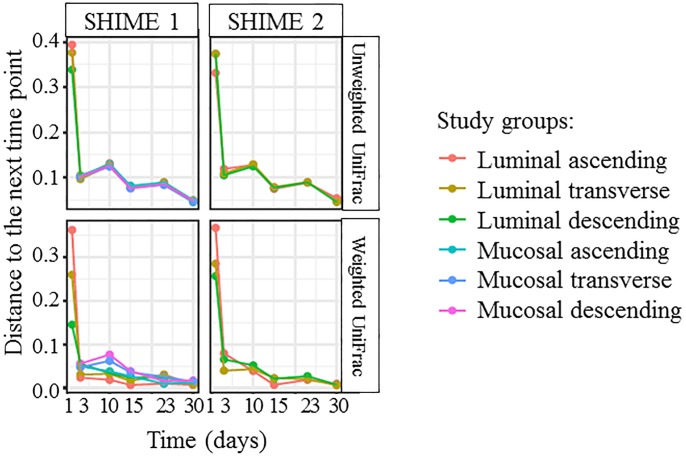
Comparison of unweighted and weighted UniFrac measurements from each time point to the next for the luminal and mucosal phases of each colon region over time.

In order to further evaluate system stability, the community structure was assessed by comparing the microbial composition of the luminal and mucosal phases in each bioreactor at each time point to the following time point, and divided by the number of days between the time points (Fig 2). From this analysis, the most changes were confirmed to occur between initiation of the experiment and day 3 post inoculation. This is apparent for both weighted and unweighted measurements. After day 10, there was little change in the community from one point in time to the next, indicated the establishment of mature, luminal and mucosal communities in each colon region. (Fig 2).

**Fig 3:**
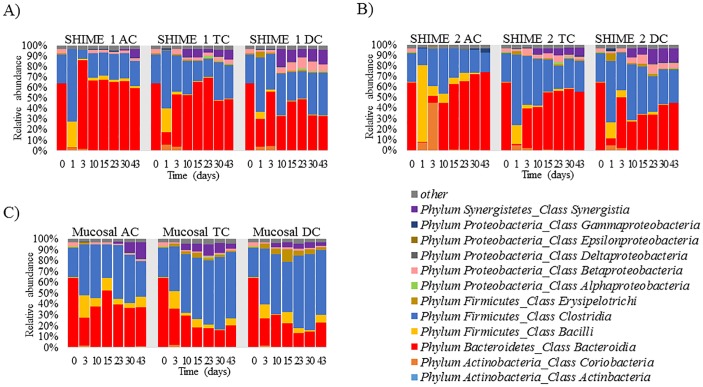
Composition of the gut microbial communities at the class level. Taxa with >1% relative abundance in samples are listed, while the rest of taxa are lumped into the “other” category. On the left of each figure, labeled 0, is the composition determined from the fecal homogenate used to inoculate the system. Communities from each region over time established in (A) SHIME 1, Luminal phase; (B) SHIME 2, Luminal phase; (C) SHIME 1, Mucosal phase.

Next, the composition of the gut microbial communities developed in both the SHIME 1 and SHIME 2 systems were determined based on the results of 16S rRNA gene sequencing, and formatted as bar graphs in terms of relative abundance at the class level (Fig 3). At time points Day 1 and 3 for the luminal phase, robust alterations in microbiota composition were observed in both SHIME 1 and SHIME 2 with taxa belonging to the *Firmicutes* phylum, namely *Bacilli* and *Clostridia*, being replaced by *Bacteroidia* (Fig 3A and 3B). Similar to the luminal phase of SHIME 1, the mucin phase demonstrated the most changes on day 3, but also reached a steady state after day 10 (Fig 3C). It was also observed that once the bacterial communities entered the steady state, the stability lasted until conclusion of the experiment. Interestingly, despite an identical inoculum being introduced into both SHIME units, there was a robust growth of Actinobacteria in the AC of SHIME 2 that was absent in the other unit. By day 10, however, Actinobacteria was largely absent in the AC of both units. From Fig 3, it was noted that some rare members in the inoculum increased to significant amounts during the experiment, such as the growth of class *Synergistia* in both the luminal and mucosal phases.

### The established luminal and mucosal gut microbial community differentiate between the three colon regions

**Fig 4:**
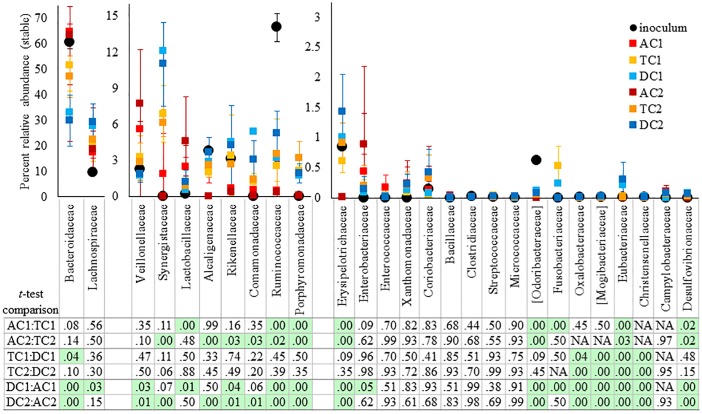
Comparison of relative abundance, at the family level, between the luminal phase of the AC, TC, and DC regions of each SHIME system after stabilization (Day10-Day 43). Only families with a greater than .01% relative abundance in at least one region were considered. The error bars represent the standard deviation between the percent abundance at these time points. The P values based on a 2 tailed student *t-*test between regions are listed in the bottom chart. The P values were corrected for multiple comparisons. A green box indicates that p < 0.05.

Since each intestinal region is maintained at a different pH value, and receives a different substrate addition (nutrients flow from AC to TC to DC), it is expected that over time the communities in these regions will differentiate. This distinct divergence between the luminal communities in the AC, TC, and DC regions can be clearly observed from Fig 4. For example, there is an apparent difference in the amount of families *Bacteroidaceae* and *Synergistaceae* between all three regions of both SHIME 1 and SHIME 2 (Fig 4). For *Eubacteriaceae*, there is a significant difference between all three regions of both SHIME systems, although this family is only present at a low abundance (Fig 4). Interestingly, there are a number of families that differ in abundance between two regions of the luminal phase, but not all three. For example, families *Ruminococcus*, *Porphyromonadaceae*, *Desulfovibrionaceae*, *Erysipelotrichaceae*, and *Odoribacteraceae* are signficantly different between the AC and TC or DC regions, but not between the TC and DC regions for both SHIME systems (Fig 4). Similiarly, families *Oxalobacteraceae*, *Mogibacteriaceae*, and *Christensenellaceae* are only statistically different for both SHIME systems between the DC and TC or AC regions, but not between the TC and AC regions (Fig 4).

**Fig 5:**
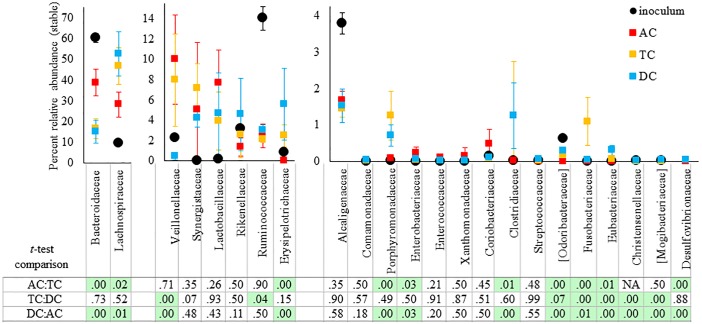
Comparison of relative abundance at the family level between the mucosal phase of the AC, TC, and DC regions of the SHIME 1 system. The relative abundance at the family level for each region at each all time points after stabilization (Day10-Day 43) were averaged and plotted together. Only families with at least a .01% relative abundance in at least one region were considered. The error bars represent the standard deviation between the percent abundance at these time points. The P values based on a 2 tailed student *t-*test between regions are listed in the bottom chart. P values were corrected for multiple comparison. A green box indicates that the p value was less than 0.05.

There was also an apparent divergence between communties of the three regions for the mucosal microbiota (Fig 5). There were only three families identified that were statistically different between all three colon regions, families *Odoribacteraceae*, *Fusobacteriaceae*, and *Eubacteriaceae*, all of which were present in low abundance. Families *Bacteroidaceae*, *Lachnospiraceae*, *Erysipelotrichaceae*, *Porphyromonadaceae*, *Enterobacteriaceae*, *Clostridaceae*, and *Desulfovibronaceae* had signficant differences between AC and TC or DC, but not between the TC and DC regions (Fig 5). On the other hand, families *Veillonellaceae*, *Christensenellaceae*, and *Mogibacteriaceae* were signficantly different between the DC and AC or TC region, but not between the AC and TC regions.

**Fig 6:**
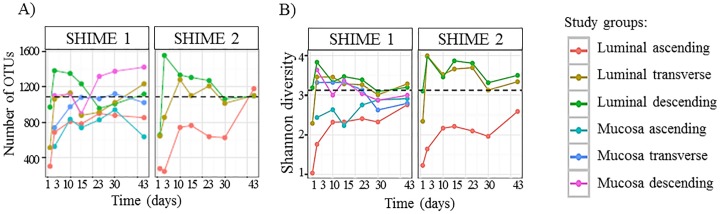
Alpha diversity measurements over time for the mucosal and luminal phase of the AC, TC, and DC regions. The black dotted line represents the Shannon diversity of the fecal sample used to inoculate the system. (A) The number of OTUs identified (B) Shannon diversity index.

The data in Figs 4 and 5 indicates that the TC region is a true intermediate between the AC and DC regions. However, according to the number of OTUs identified and the Shannon diversity index, the community developed in the TC region is more similar to the DC region in terms of diversity, and both the TC and DC communities are more similar to the fecal inoculum than the AC region (Fig 6). The AC region has a much lower number of OTUs and Shannon diversity measurement compared to the TC or DC regions and the inoculum. These observation apply to both the luminal and mucosal communities.

### The added mucosal environment allows for the colonization of specific microbial species

The results presented in Figs 1 and 4–6 suggest that the community developed in the mucosal niche for each region differs from the luminal community in terms of the abundance, and not exclusion, of specific taxa. Based on this supposition, the relative abundance at the family level for the luminal and mucosal communities for each region of SHIME 1 were analyzed and compared to each other (Table 1). Generally speaking, the mucosal environment was enriched with classes belonging to the phylum *Firmicutes*, namely classes *Clostridia*, *Bacilli*, and *Erysipelotrichi*, while a lower level of phylum *Proteobacteria* was detected (Table 1). At the family level, the mucosal environment of all three regions were statistically enriched with *Lachnospiraceae*, while lower levels of *Bacteroidaceae* were detected (Table 1), two of the most prominent families in terms of relative abundance.

**Table 1:** Percent relative abundance at the family level for the mucosal and luminal phases of each region after stabilization (greater than .01% abundance for one region). M:L refers to the mucosal abundance divided by the luminal abundance. The green shading represents families with a significant increase in mucosal abundance and pink shading represents families with a significant decrease in mucosal abundance. A * indicates that p < .05 according to a 2 tailed, student *t*-test corrected for multiple comparison.

			AC			TC			DC		
phylum	class	family	Mucosal	Luminal	M:L	Mucosal	Luminal	M:L	Mucosal	Luminal	M:L
*Firmicutes*	*Clostridia*	*Lachnospiraceae*	28.29	17.22	1.64*	46.63	20.81	2.24*	52.71	27.72	1.90*
		*Veillonellaceae*	9.99	5.58	1.79	7.96	3.22	2.47	0.48	1.72	0.28*
		*Ruminococcaceae*	2.44	0.46	5.30*	2.10	2.55	0.83	3.00	3.21	0.93
		*Clostridiaceae*	0.02	0.00	3.78	1.26	0.00	425.05*	1.25	0.01	226.23*
		*Eubacteriaceae*	0.01	0.00	56.06*	0.05	0.02	2.30	0.31	0.21	1.51
		*[Mogibacteriaceae]*	0.00	0.00	0.00	0.00	0.00	N/A	0.03	0.02	1.39
		*Christensenellaceae*	0.00	0.00	N/A	0.00	0.00	N/A	0.01	0.02	0.45
	*Bacilli*	*Lactobacillaceae*	7.66	2.48	3.09*	3.90	0.67	5.82*	4.64	0.52	8.93
		*Enterococcaceae*	0.10	0.16	0.61	0.02	0.03	0.60	0.02	0.02	0.82
		*Streptococcaceae*	0.00	0.00	1.42	0.02	0.04	0.61	0.05	0.03	2.01
		*Bacillaceae*	0.00	0.02	0.04	0.00	0.00	0.00	0.00	0.00	0.00
	*Erysipelotrichi*	*Erysipelotrichaceae*	0.01	0.00	4.25	2.47	0.60	4.08*	5.57	1.00	5.55*
*Bacteroidetes*	*Bacteroidia*	*Bacteroidaceae*	38.86	64.46	0.60*	16.58	51.38	0.32*	15.08	32.85	0.46*
		*Rikenellaceae*	1.38	0.66	2.09	2.58	3.40	0.76	4.58	4.53	1.01
		*[Odoribacteraceae]*	0.00	0.00	1.02	0.16	0.05	3.52*	0.28	0.11	2.46*
		*Porphyromonadaceae*	0.07	0.06	1.20	1.25	2.10	0.60	0.70	1.75	0.40
*Actinobacteria*	*Coriobacteriia*	*Coriobacteriaceae*	0.47	0.13	3.65	0.08	0.05	1.77	0.09	0.07	1.32
	*Actinobacteria*	*Micrococcaceae*	0.00	0.00	1.23	0.00	0.00	0.00	0.00	0.00	0.00
*Proteobacteria*	*Betaproteobacteria*	*Alcaligenaceae*	1.68	2.12	0.79	1.44	2.00	0.72	1.53	2.87	0.54
		*Comamonadaceae*	0.02	0.52	0.03	0.03	1.19	0.02*	0.04	5.40	0.01*
		*Oxalobacteraceae*	0.00	0.00	2.40	0.00	0.00	2.55	0.00	0.01	0.31
	*Deltaproteobacteria*	*Desulfovibrionaceae*	0.00	0.00	N/A	0.05	0.04	1.22	0.04	0.07	0.50
	*Epsilonproteobacteria*	*Campylobacteraceae*	0.00	0.00	N/A	0.00	0.00	N/A	0.00	0.00	N/A
	*Gammaproteobacteria*	*Enterobacteriaceae*	0.22	0.44	0.51	0.04	0.12	0.30	0.05	0.09	0.56
		*Xanthomonadaceae*	0.15	0.14	1.06	0.01	0.09	0.14	0.01	0.12	0.11*
*Fusobacteria*	*Fusobacteriia*	*Fusobacteriaceae*	0.00	0.00	7.94	1.10	0.52	2.10	0.05	0.24	0.20
*Synergistetes*	*Synergistia*	*Synergistaceae*	5.08	1.83	2.77	7.18	6.86	1.05	4.21	12.10	0.35*

There are also some differences between the luminal and mucosal communities that were region specific (Table 1). The abundance of families *Ruminococcacaeae* and *Eubacteriaceae* were signficantly higher in the mucosal phase of only the AC region; Family *Lactobacillaceae* was significantly higher in the AC and TC regions; *Families Clostridiaceae*, *Erysipelotrichaceae*, *Odoribacteraceae*, and *Comamonadaceae* were significantly different in the TC and DC regions only; *Xanthomonadaceae*, and *Synerigistaceae* were only statistically signficant between the mucosal and luminal phases in the DC region (Table 1). Taken together, these results demonstrate that the added mucosal surface allows for the development of a specific community, that this community is enriched with specific taxa, and that the composition of these communties are region dependent.

### Comparison of the community in the TWINSHIME to the original fecal inoculum

**Fig 7:**
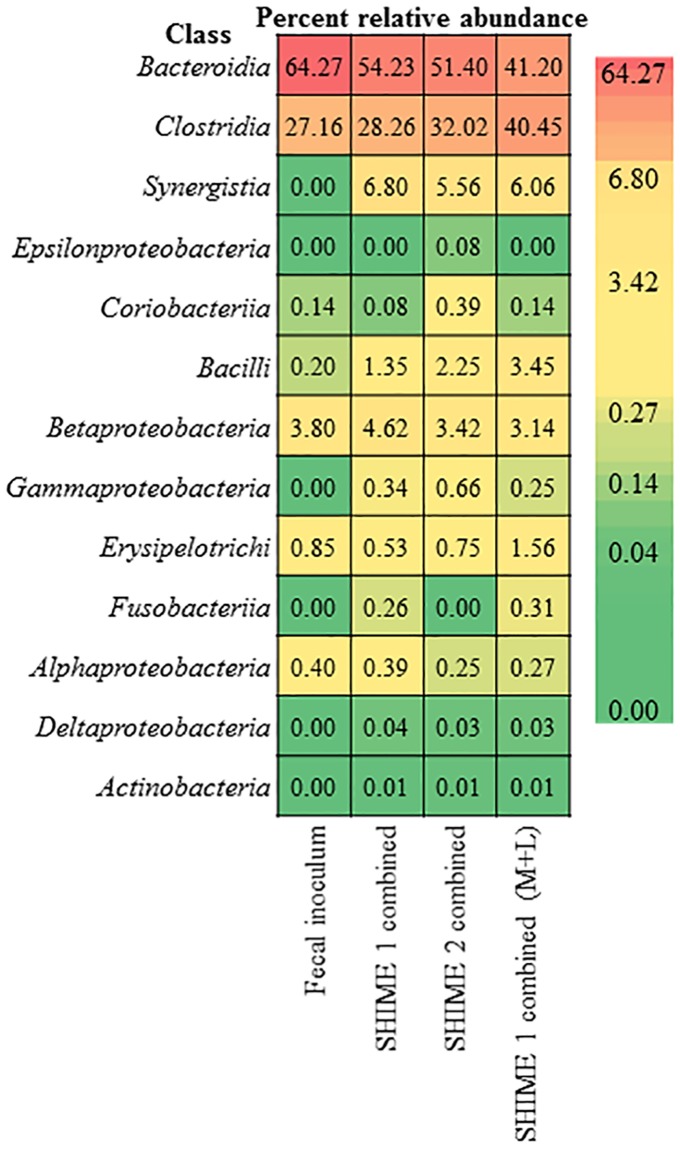
Comparison of the combined communities for each SHIME system to the fecal inoculum. SHIME 1 combined and SHIME 2 combined is the sum of the luminal phases for the AC, TC, and DC regions from day 10–43; SHIME 1 combined (M+L) is the sum of the luminal and mucosal phases of the AC, TC, and DC regions from day 10–43.

In order to consider the full community for each unit of the TWINSHIME, the stable communities of the AC, TC, and DC regions were combined together. Next, the percent relative abundance was calculated for SHIME 1 and SHIME 2, and compared to the original fecal inoculum (Fig 7). In this figure, SHIME 1 combined and SHIME 2 combined consider only the respective luminal phases, whereas SHIME 1 (M+L) considers both the luminal and mucosal phases (Fig 7).

A first, and important, observation was that all the classes detected in the inoculum were also detected in the SHIME system after stabilization. From this heat map, the following conclusions can be drawn: First, for all three systems *Bacteroidia* was the most prevalent class; yet both SHIME 1 combined and SHIME 2 combined had a ~10–12% decrease in abundance compared to the inoculum. SHIME 1 combined (M+L) has an even lower represenation of this class, due to the lower abundance of *Bacteroidia* in the mucosal community. Second, for both SHIME 1 combined and SHIME 2 combined, the second largest class is *Clostridia*, and both have similar levels compared to the fecal inoculum. Again, due to the enrichment of this class in the mucosal community, SHIME 1 combined (M+L) had a ~13% higher relative abundance of *Clostridia* compared to the original inoculum. Third, classes *Synergistia*, *Bacilli*, *Gammaproteobacteria*, *Deltaproteobacteria*, and *Actinobacteria* were more prevalant in the TWINSHIME compared to the fecal inoculum. Interestingly, *Fusobacteriia* was higher in abundance compared to the inoculum only in SHIME 1, which had a mucosal community, and *Epsilonproteobacteria* and *Coriobacteriia* are higher in abundance compared to the inoculum for SHIME 2, which had no mucosal surface. Taken together, the data presented demonstrates that the TWINSHIME is able to reproduce a human microbial community representative of the fecal inoculum. However, the developed community does not share 100% similarity to the fecal inoculum (Fig 7).

### Metabolic profile of short chain fatty acid (SCFA) production

In order to understand the metabolic activity of the gut microbiota community established in the TWINSHIME, the SCFA production was analyzed. SCFAs are the main end products of fermentation of the defined media by gut bacteria in the large colon, and represent functionality of the community. Among these saturated aliphatic organic acids, the most abundant three are acetic acid (A), propionic acid (P), and butyric acid (B), accounting for approximately 95% of the total SCFAs.

**Fig 8:**
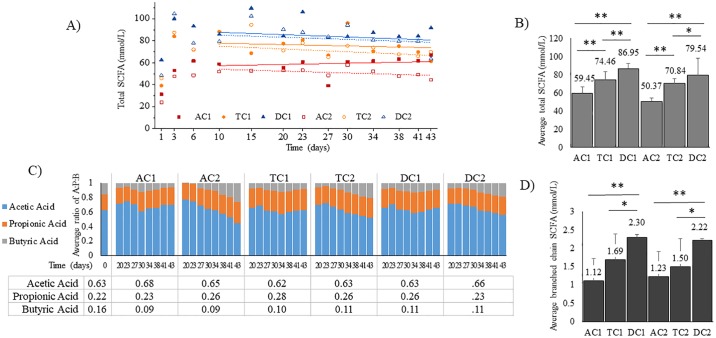
SCFA analysis for the TWINSHIME system. (A) Total SCFAs measured for each region over time (mmol/L). (B) Total average amount of SCFAs (mmol/L) and (C) Ratio of Acetic Acid: Propionic Acid Butyric Acid for each region over time. (D) Total average branched chain SCFAs (mmol/L) for each region after system stabilization (Day 17–43). The bar marked 0 is the amount of A:P:B for the original fecal inoculum. In the table underneath is the average ratio numbers for A:P:B for each region. A * indicates p< .05 and ** indicates p < .003 according to a 2-tailed student *t* test.

As shown in Fig 8A, the production of SCFA experienced vigorous variation for the first 10 days (in some cases, 15 days) after inoculation that was followed by a steady state, when the concentrations of SCFA in each bioreactor remained at the same level until the end of the experiment. This occurred by day 17 post inoculation (Fig 8A and 8C). It is also illustrated in Fig 7A that the total amount of SCFA detected in the three colonic regions increased in the sequence of AC > TC > DC. Furthermore, the concentration of SCFA detected in SHIME 1 was slightly higher than that in SHIME 2 (Fig 8B). The ratio of acetic acid and propionic acid in the total SCFA were 62–68% and 23–28%, respectively; however, the amount of butyric acid was never higher than 11% (Fig 8B and 8C). These ratios were consistent with those measured in the original fecal inoculum (Fig 8C). The total amounts of BSCFA- in each bioreactor of both SHIME systems is shown in Fig 8D. For all cases, less BSCFA were produced in AC, in comparison with DC and TC; for most time points, similar amounts of BSCFA were measured in SHIME 1 and in SHIME 2.

## Discussion

After inoculation, the fecal microbiota in the TWINSHIME competed robustly for nutrition and niches in the new environment, which required a time period to adapt to the novel conditions for the different compartments of the gastrointestinal tract. Eventually, the community reached a state in which the composition and metabolites remained constant; this is referred to as system stability. Other researchers using the TWINSHIME, following operational procedures similar to those described here, reported that it took two weeks to build a stable community [18–22], when there is 80% correlation between sequential time points. Holding this as a reference, the results from 16S rRNA sequencing indicated the cultivated community entered a steady state by day 10 post inoculation, and remained stable until the end of the experiment (Figs 1–3). System stability was also confirmed by SCFA analysis, which demonstrated that the levels of SCFA produced over time became constant, and the ratio of acetate: propionate: butyrate remained consistent (Fig 8A and 8C). Interestingly, while the community population reached a steady state by day 10 post inoculation, the SCFA data indicated that stability of microbial metabolism lagged behind, taking approximately 17 days to reach a steady state (Fig 8A). These results were consistent with previous findings [18].

One key element of the TWINSHIME is that a configuration can be established whereby the composition and function of spatially-distinct communities can be analyzed. In the case of the colon for the present study, the composition of the gut microbiota and metabolite production can be determined in both the luminal vs. mucosal phases across the ascending, transverse, and descending colons. It is expected that the communities developed in each region will be comparable to the original fecal sample in terms of composition. Yet, it is also expected that the communities in each region will be divergent from each other, based on the physiological differences between the three regions. This is consistent with the available literature [9, 33] and current opinion that the micro-environment of each colon region will promote the development of unique, colon specific communities.

The results presented in Fig 3 demonstrate that the types of bacteria present in the ascending (AC), transverse (TC), and descending (DC) regions are similar to the original fecal inoculum at the class level, consisting primarily of *Bacteroidia*, and *Clostridia*. However, Figs 4 and 5 demonstrate that the original fecal inoculum was able to differentiate into region specific sub-communities, containing unique ratios of family members. It was noted that the TC region had commonalities with both the AC and DC regions, demonstrating that the TC region acts as an intermediate between the two. This is logical considering that the TC region is physically located between the two regions, and has a median pH value.

To simulate the mucosal surface in the TWINSHIME system, mucin carriers were added to the colon regions of SHIME 1. Previous studies have shown that the mucosal-adherent microbiota differs significantly from that in the lumen *in vivo* (i.e. feces) [34, 35]. There are many reasons for this difference, including the micro-aerobic environment of the mucosal interface and the glycan-rich nature of intestinal mucus [34, 36]. According to the results presented in Fig 5 and Table 1, the mucosal carriers in the SHIME system do allow for the development of a specific community.

Similar to previously published *in vitro* results, the mucin phase had a statistically significant increase in *Lachnospiraceae* in all three regions, and *Ruminococcaceae* in the AC region (Table 1) [19]. However, since the inoculum used for the system is of human origin, it was not feasible to harvest samples from the individual colon regions of the donor at the time of donation. Therefore, there is a lack of data regarding the composition of the mucosal community for this donor *in vivo*. However, recent work looking at the mucosal surface of humans using rectal swabbing determined the mucosally associated consortium to contain both asaccharolytic and facultative genera, particularly, *Bacteroidetes Porphyromonas*, *Firmicutes Finegoldia*, *Murdochiella*, and *Peptoniphilus*, and *Proteobacteria Campylobactera* [36].

Comparatively, in the SHIME system, family *Porphyromonadaceae* was found in the mucin phase, but it was present in a lower abundance compared to the luminal phase in the TC and DC regions (Table 1). However, there is an increase in the abundance of family *Odoribacteraceae* in the mucosal phase, which is in the same class as *Porphyromonadaceae*. Similar to the *in vivo* study, in the SHIME system there was an increase in class Clostridia for the mucosal phase, although genera *Finegoldia*, *Murdochiella*, *Peptoniphilus* were not the specific ones identified. It is possible that belonging to the same phylum means that the members of these genera retain similar functional properties. It is also not always possible to identify organisms at the genera or species level using 16S sequencing. Interestingly, for the SHIME, proteobacteria was decreased in the mucin phase, indicating that there was not an enrichment in facultative bacteria (Table 1). Since the mucin carriers do not have a source of oxygenation, as the mucosal surface *in vivo* has [34], it is rational to consider that this is the reason for the difference in the presence of facultative bacteria between the *in vivo* results and the *in vitro* SHIME system. It has also been suggested that there is a shift in the microenvironment of the mucin carriers to a lower pH level, due to the accumulation of SCFAs [19]. This would also suppress growth of *Proteobacteria* taxa and promote colonization by taxa able to grow at a more acidic pH [37].

Although there may be some differences between the *in vitro* and *in vivo* mucin communities, the addition of the mucosal surface to the SHIME system is still physiologically relevant for studies of the gut microbiota. The mucin itself provides a source of nutrients for mucin-degrading microbes, and the surface allows for the attachment of microbes that may have limited mobility [19]. It is hypothesized that the communities formed on the mucin carrier interact with each other through cross feeding and cell signaling, and that there would be changes in gene regulation. However, these topics have not yet been addressed, and will be studied in the future.

Importantly, for the first time, the study presented here analyzed the mucosal communities of the TWINSHIME over the course of six weeks, addressing not only community composition for all three intestinal regions, but also confirming stability of the mucosal phase. It is interesting to see that the mucosal communities do show region specificity (Fig 5), although the difference between the TC and DC regions for the mucosal phase is not as apparent as the luminal phase (Fig 5). In fact, for the mucosal to luminal phase comparison, except for *Lactobacillaceae*, the TC region only achieved statistical difference if the DC region was also statistically different (Table 1). The DC region presented the most divergence between the luminal and mucosal phase, while conversely, the AC region had the least divergence between the two phases. In terms of stability, the mucosal and luminal phases reached a point of stability at the same time, by day 10 post inoculation, and remained here until the end of the experiment (Fig 1A and 1B).

The TWINSHIME system was designed to simulate the conditions of the human GIT for *in vitro* evaluation of the gut microbiota in response to digested food, and for many other applications. Thus, the established community of the TWINSHIME was evaluated against the donor’s sample to ensure that the system as a whole, was able to generate a community comparable to the microbiota of the human subject. This was done by combining the communities of the AC, TC, and DC regions to represent a single unit. Importantly, the luminal phase of both SHIME units led to very similar steady state configurations, suggesting that reproducible results can be achieved using SHIME technology.

As illustrated in Figs 3 and 4, classes comprising phyla *Bacteroidetes* and *Firmicutes* were the most dominant for both the donor and the SHIME systems, comprising approximately 90% of the community members. Classes in phyla *Proteobacteria*, *Actinobacteria*, *Synergistetes*, and *Fusobacteria* represented the sub-dominant components in the cultivated communities upon the systems entering the steady state. This is closely similar to the donor’s samples (Figs 3 and 4) and is consistent with the available literature [38].

While the overall composition of the community in the SHIME system was similar to that of the fecal sample, there were a few notable differences that should be addressed. Based on the analysis of 16S sequencing, there were no classes represented in the inoculum that were not also found in the SHIME system. This is important because it demonstrates that culturing the fecal sample does not result in a loss of taxa. Conversely, there are a number of classes with a higher abundance in the SHIME system compared to the fecal sample. Notably, there was a large increase in relative abundance for phylum *Synergistetes*, class *Synerigistia*, in the SHIME system, reaching a level of approximately 6% relative abundance. The presence of *Synergistia* itself is not surprising, since it was also found in the fecal inoculum, although only at 0.002% relative abundance. What is remarkable is that it grew to such a large percentage when cultured *in vitro*. Also, the level of *Synerigistia* was higher in the mucosal phase compared to the luminal phase of the AC region, demonstrated similar levels between the mucosal and luminal phases of the TC region, and was present at a higher abundance in the luminal phase then the mucosal phase of the DC region (Table 1).

Phylum *Synerigistetes* was first recognized in 2009, and contains taxa found almost ubiquitously throughout anaerobic habitats [39]. In humans, *Synergistetes* has been found on the skin, the vagina, feces, and the mouth, and is associated with anaerobic infections [39–42]. In general, the phylum has been detected primarily using culture independent methods, so there is a lack of biological characterization for this group [43]. However, the members of *Synergistetes* are all anaerobic, Gram negative, rod shaped bacteria; some species are asaccharolytic, but all ferment amino acids [43,44]. Based on this information it seems that the presence of *Synerigistia* in the SHIME system may rely on nutrient availability. It can be concluded that it is not based on the presence of mucin, since it is found in both SHIME 1 and SHIME 2 systems. In the AC region, the luminal phase would have the highest amount of sugars and starches, which would be fermented by bacteria as it moves from the AC to TC to DC. This may explain why *Synergistia*, which may be asaccharolytic and ferments amino acids, would prefer the DC region.

The increase in *Synerigistia* and other classes in the TWINSHIME compared to the fecal inoculum, is a prime example of the difference between the *in vitro* and *in vivo* systems. The increase in abundance for these specific taxa could be due to the type of nutritional input for the SHIME system. While the defined medium can be used to cultivate the gut microbiota, it cannot fully replicate an individual person’s diet. Importantly, the *in vitro* system lacks mammalian components that help shape the gut microbiota community *in vivo*, for example antibodies and antimicrobial peptides [45]. There is also a lack of absorption in the SHIME system as substrates move from one region to the next, which may contribute to the differences between the SHIME communities and the fecal sample. For inoculation and colonization of *in vitro* gut models, the steady state of the gut microbial community is the balanced result of both environmental factors and the initial qualitative diversity, but not initial quantitative balance of the fecal inoculum [12].

The community composition and population dynamics are only one part of the gut microbiota. In fact, functionality of the gut microbiota can be argued as more important than community composition. In order to fully evaluate the gut microbial community of the TWINSHIME system, the SCFA production was analyzed over time. After inoculation, the amounts and types of SCFAs produced in each colon region largely varied (Fig 8), similar to community composition. However, after entering the steady state and moving onward, the total amount of SCFA and the ratio of A : P : B remained at the same level (Fig 8A and 8D).

Despite the fact that there is more nutrition available in the AC compartment, the concentrations of SCFA increased from AC to TC, then to DC (Fig 8A–8C). This trend is opposite of that found in many *in vivo* experiments [46, 47], and can be attributed to the constant transfer of products in the TWINSHIME from upstream to downstream during fermentation, unlike in the GIT, where SCFA are absorbed and consumed. The differences in the viscosity between the ingested food in the GIT and the media of the TWINSHIME also contributes to this observed difference. For *in vivo* systems, water in the large colon is extracted, resulting in the production of an increasingly thicker slurry that inhibits mass diffusion and the interaction between gut microbiota and substrates. For the TWINSHIME, the water content in the DC is still high, similar to the AC region, and the bioreactor is under constant stirring. Therefore, mass diffusion in the DC is as convenient as in AC and TC.

As discussed in the previous section, the addition of mucosal carriers to SHIME 1 did not affect community composition or stability. However, it was noted that the total amounts of SCFA production for SHIME 1 was slightly higher than SHIME 2 throughout the experiment. This may have been contributed to the added mucosal phase, in which the bacteria are producing, and releasing SCFAs into the environment.

For BSCFA, the influence of pH also should be considered, bacterial peptidase and protease prefer neutral environments. That may explain why the BSCFA contents in the DC regions of both SHIME 1 and SHIME 2 are higher than that in the AC and TC regions (Fig 8C). Finally, the A : P : B ratio in the donor’s fecal samples of 63:22:15 matches the A : P : B ratio found from sudden death victims [47]. The ratio of A:P:B in each region of the TWINSHIME was similar to the fecal sample and remained constant after community stabilization.

In conclusion, the TWINSHIME was used to establish a gut microbial community, which entered a steady state approximately 10 days after inoculation, and remained static stable for 5 weeks, after which the experiment was terminated. The established gut microbial community maintained the original structure and composition similar to that of the donor, yet also differentiated into specific sub-communities in the three compartments representing the AC, TC, and DC of the GIT. These results confirm previous reports on TWINSHIME stability and differentiation, using more discriminative and untargeted sequencing techniques. For the first time, the mucosal community of all three regions of the TWINSHIME were analyzed and defined. The addition of mucosal carriers added another layer of complexity to the system, allowing for the stable reproduction of the gut microbiota that attaches to the mucosal surface of the GIT. The profile of SCFA production in the TWINSHIME was determined to be different than those which have been reported from *in vivo* studies, making this the major difference between the *in vitro* and *in vivo* systems. This demonstrates the impact of the colonic milieu on the functionality and kinetics of the gut microbiome.

## Supporting information

S1 Fig Graphical representation of the TWINSHIME system. The TWINSHIME system is composed of a series of bioreactors set up in sequence. Each SHIME system has a stomach (ST), small intestine (SI), ascending colon (AC), transverse colon (TC), and descending colon (DC). Three times a day the stomach is fed defined medium, after incubation for 1 hour, the ST contents are completely transferred to the SI region and pancreatic juice is added. This vessel pH is adjusted to 6.6. After 1 hour incubation, the SI contents are completely transferred to the AC region. At the same time, the pumps are turned on to move fluid from the AC to the TC, the TC to the DC, and the DC to the waste. Each colon reactor also contains mucin-agar carriers. These plastic carriers contain solidified mucin agar, and function to provide a mucosal surface on which the bacteria can grow.
(TIF)

S1 Table Average amount of SCFAs measured for each region during the experiment, presented in mmol/L concentrations. (TIF)

S2 Table 16S rRNA data: The community members for each sample were determined based on 16S rRNA gene sequencing, and grouped into indentified Operation Taxonomic Units (OTUs). In the table provided, the phylogeny for each OTU is listed on the left, and the percent relative abundance for each OTU in the samples sequenced is given.
(XLSX)

S3 Table GC_SCFA data: The short chain fatty acids (SCFAs) were quantified for each sample using GC/MS. In the table provided, the SCFA measurements for each sample are given, as well as the ratio of Acetate:Propionate:Butyrate for each sample.
(XLSX)

## References

[1] Konturek P, Haziri D, Brzozowski T, Hess T, Heyman S, Kwiecien S, et al Emerging role of fecal microbiota therapy in the treatment of gastrointestinal and extra-gastrointestinal diseases. J Physiol Pharmacol. 2015; 66: 483–91. 26348073

[2] Krishnan S, Alden N, Lee K. Pathways and functions of gut microbiota metabolism impacting host physiology. Curr Opin Biotechnol. 2015; 36: 137–45. 10.1016/j.copbio.2015.08.015 26340103 PMC4688195

[3] Maga E, Weimer B, Murray J. Dissecting the role of milk components on gut microbiota composition. Gut Microbes 2013; 4: 136–9. 10.4161/gmic.23188 23235404 PMC3595073

[4] Power S, O’Toole P, Stanton C, Ross R, Fitzgerald G. Intestinal microbiota, diet and health. Br J Nutr. 2014; 111: 387–402. 10.1017/S0007114513002560 23931069

[5] Sonnenburg J and Bäckhed F. Diet-microbiota interactions as moderators of human metabolism. Nature 2016; 6: 56–64. 10.1038/nature18846 PMC5991619 27383980

[6] Wu GD, Chen J, Hoffmann C, Bittinger K, Chen YY, Keilbaugh SA, et al Linking long-term dietary patterns with gut microbial enterotypes. Science 2011; 334:105–8. 10.1126/science.1208344 21885731 PMC3368382

[7] Nicholson JK, Holmes E, Kinross J, Burcelin R, Gibson G, Jia W, Pettersson S. Host-gut microbiota metabolic interactions. Science 2012; 336:1262–67. 10.1126/science.1223813 22674330

[8] Graf D, Di Cagno R, Fåk F, Flint HJ, Nyman M, Saarela M, Watz B.Contribution of diet to the composition of the human gut microbiota. Microb Ecol Health Dis. 2015; 26: 25656825 10.3402/mehd.v26.26164 PMC4318938

[9] Eckburg P, Bik E, Bernstein C, Purdom E, Dethlefsen L, Sargent M, et al Diversity of the Human Intestinal Microbial Flora. Science 2005; 308: 1635–38. 10.1126/science.1110591 15831718 PMC1395357

[10] Feria-Gervasio D, Tottey W, Gaci N, Alric M, Cardot J, Peyret P, et al Three-stage continuous culture system with a self-generated anaerobia to study the regionalized metabolism of the human gut microbiota. J Microbiol Methods 2014; 96: 111–8. 10.1016/j.mimet.2013.11.015 24333608

[11] Guerra A, Etienne-Mesmin L, Livrelli V, Denis S, Blanquet-Diot S, Alric M. Relevance and challenges in modeling human gastric and small intestinal digestion. Trends Biotechnol 2012; 30: 591–600. 10.1016/j.tibtech.2012.08.001 22974839

[12] Payne A, Zihler A, Chassard C, Lacroix C. Advances and perspectives in in vitro human gut fermentation modeling. Trends Biotechnol. 2012; 30: 17–25. 10.1016/j.tibtech.2011.06.011 21764163

[13] Molly K, De Smet I, Nollet L, Vande Woestyne M, Verstraete W.Effect of Lactobacilli on the Ecology of the Gastro-intestinal Microbiota Cultured in the SHIME Reactor. Microb Ecol Health Dis. 1996; 9: 79–89.

[14] Molly K, Vande Woestyne M, De Smet I, Verstraete W. Validation of the Simulator of the Human Intestinal Microbial Ecosystem (SHIME) Reactor Using Microorganism-associated Activities. Microb Ecol Health Dis. 1994; 7: 191–200.

[15] Macfarlane GT, Cummings JH, Macfarlane S, Gibson GR. Influence of retention time on degradation of pancreatic-enzymes by human colonic bacteria grown in a 3-stage continuous culture system. J Appl Bacteriol. 1989; 67:521–527. 2480341

[16] Miller TL, Wolin MJ. Fermentation by the human large-intestine microbial community in an in vitro semi continuous culture system. Appl Environ Microbiol. 1981; 42:400–07. 7027952 10.1128/aem.42.3.400-407.1981 PMC244027

[17] Minekus M, Smeets-Peeters M, Bernalier A, Marol-Bonnin S, Havenaar R, Marteau P. A computer-controlled system to stimulate conditions of the large intestine with peristaltic mixing, water absorption and absorption of fermentation products. Appl MIcrobiol Biotechnol. 1999; 53:108–14. 10645630 10.1007/s002530051622

[18] Possemiers S, Verthe K, Uyttendaele S, Verstraete W. PCR-DGGE-based quantification of stability of the microbial community in a simulator of the human intestinal microbial ecosystem. FEMS Microbiol Ecol. 2004; 49:495–507 10.1016/j.femsec.2004.05.002 19712298

[19] Van den Abbeele P, Grootaert C, Marzorati M, Possemiers S, Verstraete W, Gérard P, et al Microbial community development in a dynamic gut model is reproducible, colon region specific, and selective for Bacteroidetes and Clostridium cluster IX. Appl Environ Microbiol. 2010; 76: 5237–46. 10.1128/AEM.00759-10 20562281 PMC2916472

[20] Van de Wiele T, Van den Abbeele P, Ossieur W, Possemiers S, Marzorati M. 2015, p 3005–17. The Simulator of the Human Intestinal Microbial Ecosystem (SHIME) *In* Verhoeckx K, Cotter P, López-Expósito I, Kleiveland C, Lea T, Mackie A, Requena T, Swiatecka D, and Wichers H. The Impact of Food Bioactives on Health-in vitro and ex vivo models. SpringerLink. 29787039

[21] Vigsnaes LK, Van den Abbeele P, Sulek K, Frandsen HL, Steenhold C, Brynskov J, et al Microbiotas from UC patients display altered metabolism and reduced ability of LAB to colonize mucus. Sci Rep. 2012; 3:e1–10. 10.1038/srep01110 PMC3552269 23346367

[22] Van den Abbeele P, Belzer C, Goosens M, Kleerebezem M, De Vos WM, Thas O, et al Butyrate-producing *Clostridium* Cluster XIVa species specifically colonize mucins in an in vitro gut model. ISME J 2013; 7: 949–61 10.1038/ismej.2012.158 23235287 PMC3635240

[23] Van Herreweghen F, Van den Abbeele P, De Mulder T, De Weirdt R, Geirnaert A, Hernandez-Sanabria E, et al In vitro colonization of the distal colon by *Akkermansia muciniphila* is largely mucin and pH dependent. Beneficial Bacteria 2017; 8:81–95. 10.3920/BM2016.0013 27824274

[24] Wang M, Firrman JA, Zhang L, Arango-Argoty G, Tomasula P, Liu L, Xiao W, Yam K. Apigenin Impacts the Growth of the Gut Microbiota and Alters the Gene Expression of *Enterococcus*. Molecules 2017; 22: e1–22. 10.3390/molecules22081292 PMC6152273 28771188

[25] Caporaso JG, Kuczynski J, Stombaugh J, Bittinger K, Bushman F, Costello E, et al QIIME allows analysis of high-throughput community sequencing data. Nat Methods 2010; 7: 335–6. 10.1038/nmeth.f.303 20383131 PMC3156573

[26] Edgar R. Search and clustering orders of magnitude faster than BLAST. Bioinformatics 2010; 26: 2460–1. 10.1093/bioinformatics/btq461 20709691

[27] Cole J, Wang Q, Cardenas E, Fish J, Chai B, Farris R, et al The Ribosomal Database Project: improved alignments and new tools for rRNA analysis. Nucleic Acids Res. 2009; 37(Database issue): D141–5. 10.1093/nar/gkn879 19004872 PMC2686447

[28] McDonald D, Price M, Goodrich J, Nawrocki E, DeSantis T, Probst A, et al An improved Greengenes taxonomy with explicit ranks for ecological and evolutionary analyses of bacteria and archaea. ISME J 2012; 6: 610–8. 10.1038/ismej.2011.139 22134646 PMC3280142

[29] Lozupone C, Knight R. UniFrac: a new phylogenetic method for comparing microbial communities. Appl. Environ. Microbiol. 2005; 71:8228–35. 10.1128/AEM.71.12.8228-8235.2005 16332807 PMC1317376

[30] Lozupone C, Hamady M, Kelley S, Knight R. Quantitative and qualitative ß diversity measures lead to different insights into factors that structure microbial communities. Appl. Environ. Microbiol. 2007; 73:1576–85. 10.1128/AEM.01996-06 17220268 PMC1828774

[31] Benjamini Y, Hochberg Y. Controlling the false discovery rate: a practical and powerful approach to multiple testing. J Royal Stat. Soc. 1995; 57:289–300.

[32] García-Villalba R, Giménez-Bastida J. A, García-Conesa M. T, Tomás-Barberán F. A, Espín J. C, Larrosa M. Alternative method for gas chromatography-mass spectrometry analysis of short-chain fatty acids in faecal samples. J. Sep. Sci. 2012; 35:1906–1913. 10.1002/jssc.201101121 22865755

[33] Fan P, Li L, Rezaei A, Eslamfam S, Che D, Ma X. Metabolite of dietary proteins by intestinal microbes and their impact on gut. Curr Protein Pept Sci. 2015; 16:646–54. 26122784 10.2174/1389203716666150630133657

[34] Albenberg L, Esipova T, Judge C, Bittinger K, Chen J, Laughlin A, et al Correlation between intraluminal oxygen gradient and radial partitioning of intestinal microbiota in humans and mice. Gastroenterology 2014; 147:1055–63. 10.1053/j.gastro.2014.07.020 25046162 PMC4252572

[35] Eckburg PB, Bik EM, Bernstein CN, Purdom E, Dethlefsen L, Sargent M, et al Diversity of the human intestinal microbial flora. Science 2005; 308: 1635–8. 10.1126/science.1110591 15831718 PMC1395357

[36] Li H, Limenitakis JP, Fuhrer T, Geuking MB, Lawson MA, Wyss M, et al The outer mucus layer hosts a distinct intestinal microbial niche. Nat Commun. 2015; 22:8292–8305. 10.1038/ncomms9292 PMC4595636 26392213

[37] Duncan S, Louis P, Thomson J, Flint H. The role of pH in determining the species composition of the human colonic microbiota. Environ Microbiol. 2009; 11:211–22. 10.1111/j.1462-2920.2009.01931.x 19397676

[38] The Human Microbiome Project Consortium. Structure, Function and Diversity of the Healthy Human Microbiome. Nature 2012; 486:207–14. 10.1038/nature11234 22699609 PMC3564958

[39] Jumas-Bilak E, Roudiere L, Marchandin H. Description of ‘Synergistetes’ phyl. Nov. and emended description of the phylum ‘Defierribacteres’ and of the family Syntrophomonadaceae, phylum ‘Firmicutes’. Int J Syst Evol Microbiol. 2009; 59:1028–35. 10.1099/ijs.0.006718-0 19406787

[40] Vartouklan S, Pamer R, Wade W. Cutlivation of a *Synergistetes* strain representing a previously uncultivated lineage. Environ Microbiol. 2010; 12:916–28. 10.1111/j.1462-2920.2009.02135.x 20074237 PMC2916210

[41] Baumgartner A, Thurnheer T, Luthi-Schaller H, Gmur R, Belibasakis N. The phylum *Synergistetes* in gingivitis and necrotizing ulcerative gingivitis. J Med Microbiol. 2012; 61:1600–09. 10.1099/jmm.0.047456-0 22878253

[42] Davis C, Webb R, Sly L, Denman S, McSweeney C. Isolation and survey of novel fluoroacetate-degrading bacteria belonging to the phylum *Synergistetes*. FEMS Microbiol Ecol. 2012; 80:671–84. 10.1111/j.1574-6941.2012.01338.x 22372434

[43] Bhandari V, Gupta R. Molecular signatures for the phylum Synergistetes and some of its subclades. Anotonie van Leeuwenhoek 2012; 102: 517–40. 10.1007/s10482-012-9759-2 22711299

[44] Marchandin H, Damay A, Roudiere L, Teyssier C, Zorgniotti I, Dechaud H, et al Phylogeny, diversity and host specialization in the phylum *Synergistetes* with emphasis on strains and clones of human origin. Research in Microbiology 2010; 161: 91–100. 10.1016/j.resmic.2009.12.008 20079831

[45] Kato L, Kawamoto S, Maruya M, Fagarasan S. The role of the adaptive immune system in regulation of gut microbiota. Immunol Rev. 2014; 260: 67–75. 10.1111/imr.12185 24942682

[46] den Besten G, van Eunen K, Groen AK, Venema K, Reijingoud DJ, Bakker BM. The role of short-chain fatty acids in the interplay between diet, gut microbiota, and host energy metabolism. J. Lipid Research 2013; 54: 2325–39. 23821742 10.1194/jlr.R036012 PMC3735932

[47] Cummings JH, Pomare EW, Branch WJ, Naylor CP, Macfarlane GT. Short chain fatty acids in human large intestine, portal, hepatic and venous blood. Gut 1987; 28:1221–27.1. 3678950 10.1136/gut.28.10.1221 PMC1433442

