# Supplementary figures and images for: Establishing a mucosal gut microbial community in vitro using an artificial simulator
Source: PLoS One. 2018 Jul 17;13(7):e0197692. doi: 10.1371/journal.pone.0197692 (PMC6050037; doi:10.1371/journal.pone.0197692)

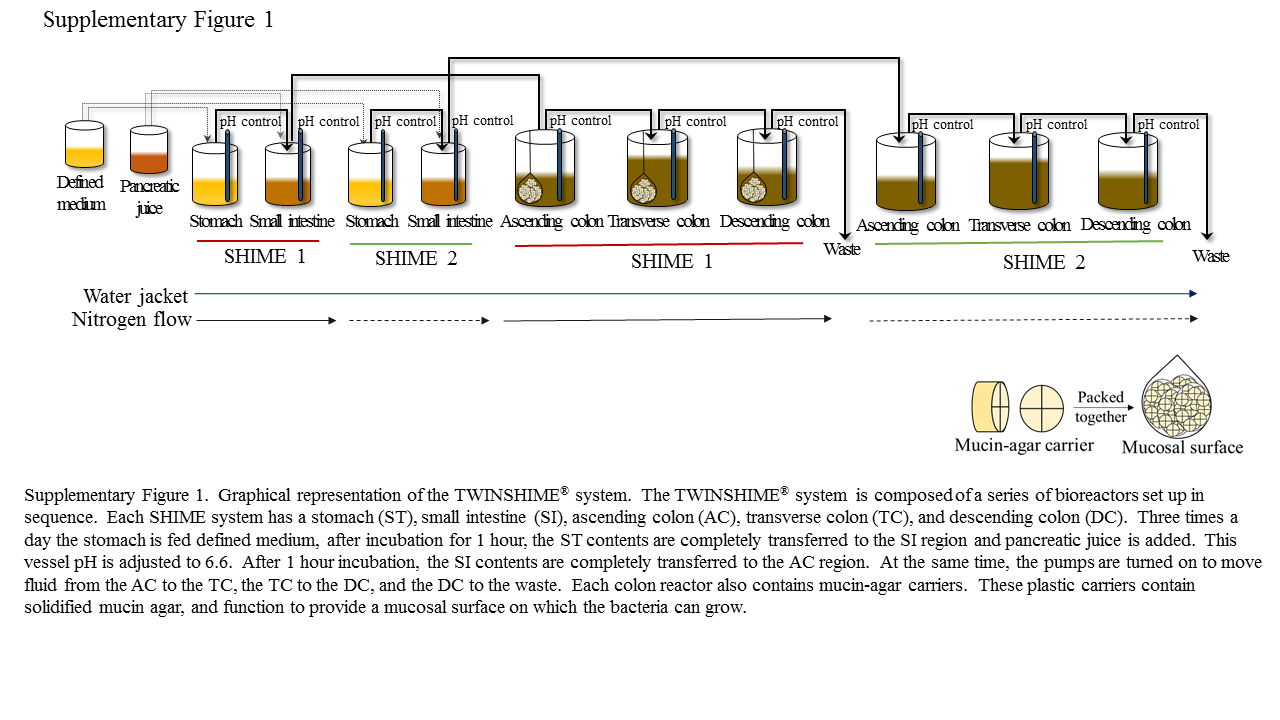

Supplement: S1 Fig — The TWINSHIME system is composed of a series of bioreactors set up in sequence. Each SHIME system has a stomach (ST), small intestine (SI), ascending colon (AC), transverse colon (TC), and descending colon (DC). Three times a day the stomach is fed defined medium, after incubation for 1 hour, the ST contents are completely transferred to the SI region and pancreatic juice is added. This vessel pH is adjusted to 6.6. After 1 hour incubation, the SI contents are completely transferred to the AC region. At the same time, the pumps are turned on to move fluid from the AC to the TC, the TC to the DC, and the DC to the waste. Each colon reactor also contains mucin-agar carriers. These plastic carriers contain solidified mucin agar, and function to provide a mucosal surface on which the bacteria can grow. (TIF) [file pone.0197692.s001.TIF]

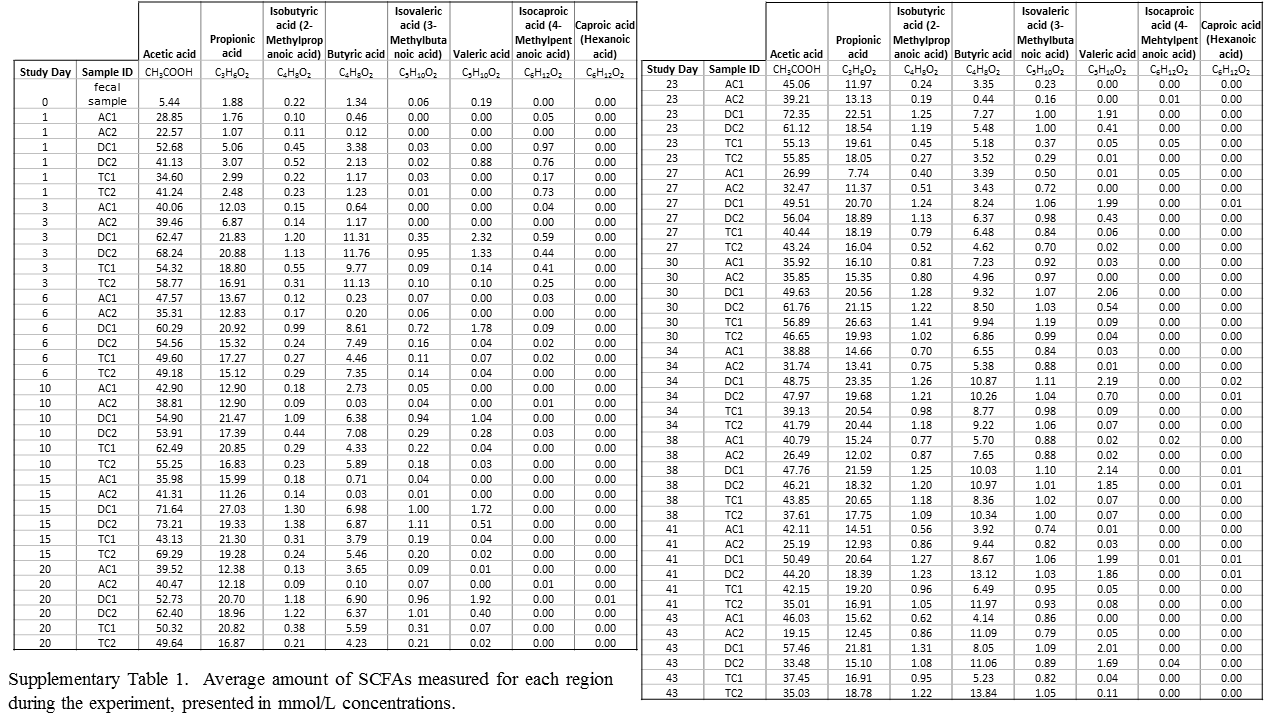

Supplement: S1 Table — (TIF) [file pone.0197692.s002.TIF]
